# Leveraging Vision Attention Transformers for Detection of Artificially Synthesized Dermoscopic Lesion Deepfakes Using Derm-CGAN

**DOI:** 10.3390/diagnostics13050825

**Published:** 2023-02-21

**Authors:** Misaj Sharafudeen, Andrew J., Vinod Chandra S. S.

**Affiliations:** 1 Department of Computer Science, University of Kerala, Kerala 695581, India; 2 Department of Computer Science and Engineering, Manipal Institute of Technology, Manipal Academy of Higher Education, Manipal 576104, India

**Keywords:** artificial synthesis, medical deepfakes, dermoscopic skin lesions, generative adversarial networks, attention vision transformers

## Abstract

Synthesized multimedia is an open concern that has received much too little attention in the scientific community. In recent years, generative models have been utilized in maneuvering deepfakes in medical imaging modalities. We investigate the synthesized generation and detection of dermoscopic skin lesion images by leveraging the conceptual aspects of Conditional Generative Adversarial Networks and state-of-the-art Vision Transformers (ViT). The Derm-CGAN is architectured for the realistic generation of six different dermoscopic skin lesions. Analysis of the similarity between real and synthesized fakes revealed a high correlation. Further, several ViT variations were investigated to distinguish between actual and fake lesions. The best-performing model achieved an accuracy of 97.18% which has over 7% marginal gain over the second best-performing network. The trade-off of the proposed model compared to other networks, as well as a benchmark face dataset, was critically analyzed in terms of computational complexity. This technology is capable of harming laymen through medical misdiagnosis or insurance scams. Further research in this domain would be able to assist physicians and the general public in countering and resisting deepfake threats.

## 1. Introduction

As the name implies, deepfakes employ artificial intelligence and deep learning to manipulate or generate inexistent visual or audio content. The breakthroughs in deepfake generation offer both benefits and drawbacks. The 2019 video footage of former US President Barack Obama, where he was faked into improper usage of language, was released as public awareness of the would-be-weapon of the near future by filmmaker Jordan Peele [1]. The outreach of the terminology deepfakes hit its maximum when video footage of Facebook CEO Mark Zuckerberg announcing the closure of Facebook to the public went viral, which was a deep fake. Though the concept of forging and manipulating visual content is not new, the advent of highly realistic indistinguishable fake content is quite challenging since they call for efficient models for their detection. The face is a person’s most distinguishing characteristic. Face modification poses a growing security issue due to the rapid advancement of face synthesis technology. Frequently, people’s faces can be replaced with those of others who seem real.

We tend to trust what we see. The common public is well aware of generating fake images through easily accessible software such as Photoshop. However, we are yet to be informed about the possibilities of generating fake videos and their convincing nature due to their highly realistic output. Deepfake content is disseminating more quickly than ever in the twenty-first century due to the growth of multiple social networking sites, making it a global threat. It explains how deepfake technology could be one of the digital weapons facing future generations, producing extremely unacceptable ethical, moral, and legal concerns [2]. Through digital impersonation, it would be considered easy to cyberattack a person, a public figure, or a cause. Moreover, convenient and discrete public access to digital content can only elevate the effects. On the other hand, deepfake technology has a brighter side to the positive application in the entertainment industry. There may soon come a time when an eligible actor’s physical looks and vocals may be deep faked and inserted into video recordings of films acted out by another talented individual. Deepfakes are cutting-edge technology that eventually produces smart applications that can enable someone to be a part of the trend. The traditional image forgery detection approaches have yet to prove efficient in detecting deepfake content.

The recent inclination of deepfake research can be split into two main categories: (1) Deepfake generation, which focuses on synthesizing and improving existing state-of-the-art techniques with respect to computational complexities and training time, and (2) Deepfake detection, which concentrates on developing reliable and universal classifiers that can be deployed in the wild.

The promise of current deepfake generation and detection research lies solely in facial deepfake recognition. There are various datasets containing millions of images for face recognition tasks, which are utilized for face deepfake synthesis. The Visual Geometry Group Face dataset (VGGFace) and the CelebFaces Attributes Dataset (Celeb-A) are huge datasets comprising over 200,000 images. The deciphering gap, however, largely potentiates medical deepfake synthesis and detection that should call for much attention.

Extensive research is being encouraged in medical diagnostics, and disease detection using machine learning, deep learning, and ensemble techniques [3,4,5]. Pre-trained networks such as MobileNet and EfficientNets have been preferred over handcrafted features for healthcare diagnosis due to their capacity to adapt to new data by transferring the learned representations from one domain to another [6,7]. Given that medical fakes pose a future hazard, the identification of medical deepfakes prior to a medical diagnosis would only be extremely intuitive. In the case of manipulated facial data, the artificial visual irregularities in the skin tone were initially recognizable to the human eye [8]. On the contrary, new and improved modified GANs are still being released in rapid succession.

### 1.1. Generation of Synthesized Images

Depending on the degree of alteration, synthesized images may be divided into three categories: face-swapping, face-reenactment or attribute manipulation, and inexistent whole face synthesis [9]. Face-swap is a technique where the subject’s face from the source image is automatically swapped out with that of the subject from the target face. Face-reenactment is the manipulation of facial expressions, such as adding the attributes of a source person, which includes eyes, emotions, and facial features, onto an output image. Face generation aims to create lifelike representations of a human face that might or might not exist in reality. The ability of human eyes to distinguish between fake and genuine content has become increasingly challenging due to the high quality of these synthetic images. Table 1 briefs the state-of-the-art in each of the generation categories.

The basis of synthesized multimedia is the mathematical formulations of Generative Adversarial Networks (GANs) [10]. Deepfakes are generated using several variants of GANs by generating new samples that imitate an existing data set. In the vanilla GAN model, a low-dimension random noise is transformed into photorealistic images using the adversarial training behavior of a generative model and the classification nature of a discriminative model. Briefly, while the generator trains its network to generate realistic fake content from a set of training images, the discriminator distinguishes an incoming image as real or fake.

(1) $$L_{G}=min\left[\frac{1}{n}\sum_{i=1}^{n}\left(log\left(1-D\left(G\left(z_{i}\right)\right)\right)\right)\right]$$

(2) $$L_{D}=max\left[\frac{1}{n}\sum_{i=1}^{n}\left(logD\left(x_{i}\right)+log\left(1-D\left(G\left(z_{i}\right)\right)\right)\right)\right]$$

where *z* is the random noise vector, $x_{i}$ is the training samples from the real dataset, and $z_{i}$ are the generated fake datapoints. Technically, *z* gets molded into highly realistic images $z_{i}$ by the model in a min-max pull of the cost functions of generator $L_{G}$ and discriminator $L_{D}$ (Equations (1) and (2)). Here, The generator attempts to reduce the likelihood that the discriminator will accurately categorize images, and the discriminator tries to maximize the probability of its efficient classification where real images are classified as real and fake images are classified as fake [11].

**Table 1:** Overview of Image Generation Models.

Reference	Mode	Model	Data	Synthesized Quality
*Korshunova et al.* [12]	Face Swap	GAN	VGGFace	256 × 256
*Natsume et al.* [13]	Face Swap	GAN	CelebA	128 × 128
*Li et al.* [14]	Face Reeanctment	GAN	VGG Face CelebA	256 × 256
*Kim et al.* [15]	Face Reenactment	cGAN	customized	1024 × 1024
*Liu et al.* [16]	Face Synthesis	CoGAN	CelebA	64 × 64
*Karras et al.* [17]	Face Synthesis	PGGAN	CelebA	1024 × 1024
*Karras et al.* [18]	Face Synthesis	StyleGAN	ImageNet	1024 × 1024
*Brock et al.* [19]	Face Synthesis	BigGAN	ImageNet	512 × 512
*Frid-Adar et al.* [20]	CT images	DCGAN	Own data	64 × 64
*Thambawita et al.* [21]	ECG	WaveGAN	Own data	10 s ECG
*Mirsky et al.* [22]	CT Lung Nodules	CTGAN	LIDC-IDRI	3 × 64 × 64

An enhanced deepfake generation method employing GAN was suggested in [12], which added a perceptual loss to the VGGface synthesis. They created texture-less, smooth images. Natsume et al. [13] employed two different GANs to encode the latent dynamics of facial and hair attributes. However, the approach was sensitive to occlusions and lighting effects. In order to maintain the desired properties such as stance, expression, and occlusion, Li et al. [14] produced facial images by employing two real face datasets. Nevertheless, the imperfections created during synthesis were projected because of the stripping effect and inadequate resolution.

Instead of only changing the target individual’s facial expression, in [15], GANs were conditioned using a conditional GAN (cGAN) to mimic human expressions, including blinking and smiling. In [23], GANs were then effectively employed for face synthesis by integrating a perceptual loss with conditional GANs. The resolutions, however, remained poor compared to real-face photographs. Instead of training only one GAN, Liu et al. [16] suggested Coupled GAN (CoGAN), where each of the two GANs was in charge of synthesizing images in a specific domain. Low picture resolution is a challenge for the majority of these deep learning-based image synthesis approaches. Karras et al. [17] proposed the Progressive Growing GANs (PG-GAN) to demonstrate high-quality face synthesis with enhanced image quality by progressively adding layers to the networks during the training process. Checker effects and blob-like effects were quite common in the aforementioned methods of synthesis, leaving visible traces of manipulation. In [18], PGGANs were improved to propose Style GAN by learning to transform a latent noise (Z) to an intermediate latent vector (W), rather than mapping latent code z to an image resolution, as in the vanilla GAN architecture. This controlled different visual characteristics to be transferred to another domain. The BigGAN architecture [19] used residual networks and an increased batch size to improve resolution.

Recently, GANs have been used to create deep medical fakes and machine learning-based diagnosis tools to trick medical professionals by erasing or adding symptoms and signs of medical illnesses. However, this method was largely used to provide more medical data for study and research innovations. A Deep Convolutional GAN (DCGAN) was suggested by Frid-Adar et al. to synthesize high-definition CT (Computed Tomography) images [20]. The artificial creation of brain tumors, cancerous cell structures, and challenging-to-reproduce histopathological data is suggested [24,25,26]. The generation of complicated Electrocardiograms (ECG) using a WaveGAN was suggested by Thambawita et al. [21].

Recently, the technology has been made available to anybody interested in creating new data for a positive study. Indeed, synthetic data has piqued the interest as a potential road ahead for increased reproducibility in research. However, this technology’s detection has yet to be extensively investigated, and it may become a weapon in the medical arena in the future. The Jekyll framework was the first to demonstrate a style transfer mechanism for medical deepfake attacks in X-rays and retinal fundus modalities [27]. A conditional generative adversarial network (cGAN) called CT-GAN was developed by Mirsky et al. to add or remove malignant nodules from lung CT data that over 90% of the clinicians failed to spot [22]. The motivations and reasons for such attacks could be many, for instance, fabricating research, a misdiagnosis on falsified medical data leading to permanent physical or mental effects on patients due to wrong medications, and even insurance frauds claiming huge payouts.

### 1.2. Detection of Synthesized Images

Existing approaches target either the spatial inconsistencies left during the generation or are based on pure content classification. The spatial artifacts include background artifacts and GAN fingerprints. Deep neural models can capture intrinsic characteristics and, thus, are used in data-driven techniques to classify and identify modifications. On studying several existing deep neural network models for the detection of deepfake attacks, we observed that most researches are presented by generating their own dataset.

The scientific community had forecasted the threats involved with the advent of GANs and had come up with open-sourced datasets such as the Deepfake Detection Challenge (DFDC) [28], Diverse Fake Face Dataset (DFFD) [29], FaceForensics++ [30] and many more. Research on detection mechanisms is mostly focused on exploring the pre-trained models so as to leverage already learned feature maps onto a new domain. This seems to work well with self-synthesized datasets rather than benchmarked ones. In [31], an ensemble of EfficientNets was fine-tuned on DFDC to achieve results comparable to the challenge-winning team. However, the winning solution could only achieve an accuracy of ∼65%. A light weighted CNN was proposed in [32] with as much as only two and three convolution layers. On the DFDC data, their model outperformed the state-of-the-art VGG-19, Inception-ResNet-v2, and Xception Networks. Suganthi et al. [33] proposed a statistical approach where fisher faces were extracted from texture components using the local binary pattern algorithm. A Deep Belief Network (DBN) could classify the DFFD dataset with 88.9% sensitivity and 93.76% specificity.

Since medical deepfakes are fairly recent, few detection techniques have been used to lessen their impact. On CT-GAN-produced data, Solaiyappan et al. tested numerous machine learning and pre-trained Convolution Neural Networks (CNNs) [34]. Limited data and model simplicity both had a negative impact on the success of detection. The detection rate of the models was quite low when the experiments were conducted as a multi-class categorization of tampered versus untampered injected and removed nodules. The various pre-trained networks attained a maximum of 80% classification accuracy when considering the DenseNet121 variant. In [35], we learned a more sophisticated 3-dimensional neural architecture on localized nodules from CT-GAN generated data and could attain a marginal accuracy gain of over 10%. The temporal feature extraction across multiple slices performed by a 3DCNN had more significance than the spatial content learning of individual slices. This led us to think that utilizing Vision Transformers to leverage the attention processes weighing the relevance of each element of the input data separately could replace the feature learning procedures through convolutions [36].

### 1.3. Motivation

**Figure 1:**
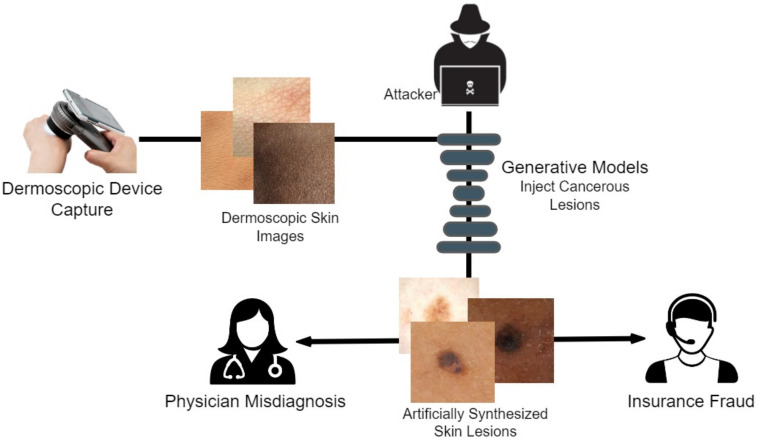
Behaviour of an attacker for Dermoscopic fakes to be used for the discrepancy.

We opted to research the dermoscopic avenue of medical deepfakes, as this modality is the easiest technique for capturing skin cancer diagnosis data due to easier targeted attacks. Figure 1 illustrates how an attacker can easily manipulate healthcare and other biomedical imagery. Dermoscopic devices are standard handheld, non-invasive machines capable of capturing high-resolution skin images. Most often, skin-prone diseases are initially diagnosed by a physician from these images. Using a generative framework, a black hat expert could easily maneuver different skin cancers from mere human skin image samples. The generative model could either generate new fake lesions or transform existing non-dangerous tumors into late-stage malignant lesions. The current healthcare system is designed to provide insurance schemes based on a doctor’s diagnosis and biomedical imaging modalities as proof. Consequently, both the physician and the inspection agent at the insurance end are likely to believe the attacker’s fallacy of tampering and manipulating the medical images during the inquiry and diagnostic stages.

With this in the lead, we propose a modified conditional GAN named Derm-CGAN to generate high-definition dermoscopic images of skin lesions. Analyzing the synthesized data with real cancerous data reveals high resemblance and realism. We compute the Representation Similarity Matrix (RSM) to project the resemblance. Further, the state-of-the-art Vision Transformers (ViT) are explored in the feature learning and categorization of real and fake dermoscopic data. The best-performing ViT configuration was further analyzed by testing on synthesized face images from the DFFD dataset as well as on selected pre-trained networks to consolidate the findings.

The novelty of the research work is contributed as:

- Designed a dermatology-conditioned generative adversarial network named Derm-CGAN for the artificial synthesis of dermoscopic images.
- A similarity analysis technique is illustrated that compares the realism of deepfakes to genuine data.
- Proposed an architecture for dermoscopic deepfake image detection based on a modified vision attention transformer.
- Critical analysis has been performed on the detection mechanism in Diverse Fake Face Dataset (DFFD) and state-of-the-art pre-trained networks.

## 2. Materials and Methods

**Figure 2:**
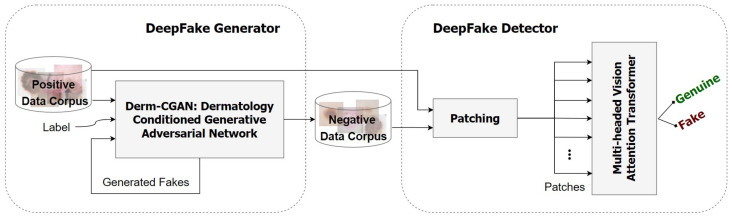
Overall structure of the proposed framework.

The general architecture of the proposed framework is illustrated in Figure 2. We observed a need for publicly available synthesized data in medical deepfake detection. For this reason, the Derm-CGAN was developed as a modified version of the current Conditional GAN-based image translation frameworks by training the network continuously until it reached a stable momentum generating highly realistic fake content [37]. The negative data corpus is a collection of synthesized skin lesions by Derm-CGAN. A Multi-headed Vision Attention Transformer (ViT) was then trained on the real dermoscopic conditions (positive dataset) as well as the counterfeited lesions (negative dataset). The network extracts latent representations from patches of dermoscopic images to determine if the incoming input is genuine or fraudulent.

Derm-CGAN defines fake skin cancer data generation as an image-to-image translation challenge. Dermoscopy devices capture dermoscopic images that enhance the visualization of the deeper layers of the skin. Recreating high-definition dermoscopic data seems arduous due to the complex structures and tissue detailing of human skin, including human hair follicles and color variations. Our model learns explicitly to discover a function mapping containing a pre-specified skin condition and the underlying skin attributes, such as the size and color of skin disorders.

### 2.1. Positive Dataset

We utilized the well-known ISIC2019 dataset of eight separate skin lesion categories for the real bonafide data. The International Skin Imaging Collaboration (ISIC) datasets are the largest known repository of skin lesions collected from clinics around the world. ISIC2019 originally comprises 25,331 images assembled from the HAM10000 [38], BCN20000 [39], and MSK [40] datasets, each of which is a standard collection of dermoscopic skin lesion images gathered from reputed cancer centers around the world. The repository includes benign as well as malignant skin cancer images. Benign classes are the subdued forms of cancer that may turn hazardous if left untreated, whereas malignant cancers are potentially dangerous and may even lead to life-threatening situations.

We chose 600 images each from six different skin lesion categories as the bonafide dataset. The benign classes selected were Actinic keratosis (AKIEC), Benign keratosis (BKL), Melanocytic nevus (NEVI), and Vascular lesions (VASC). Two malignant categories, Basal cell carcinoma (BCC) and Melanoma (MEL) classes of lesions, were chosen to maintain divergence in the positive data corpus. The classes with the least representation (fewer than 600 in total), notably Dermato Fibroma (DF) and Squamous Cell Carcinoma (SCC), were omitted from consideration as they would cause inconsistencies during the training of the Derm-CGAN.

### 2.2. Negative Dataset

**Figure 3:**
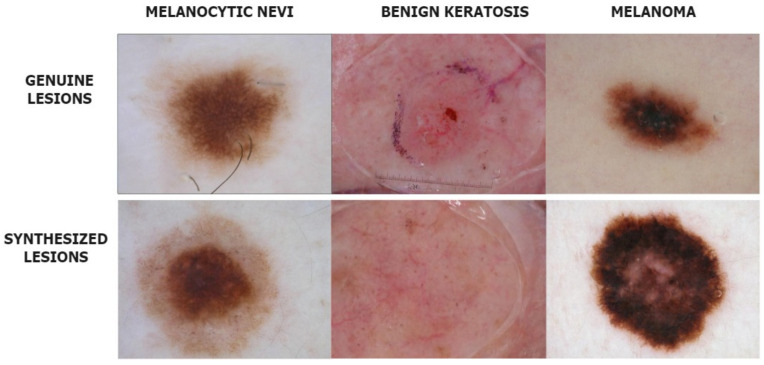
Instances from the Real and Deepfake dermoscopic datasets.

The deepfake dermoscopic dataset was prepared using the novel Derm-CGAN architecture we engineered as an extension of conditional GANs (cGAN). Conditional GANs generate images using a random latent vector and corresponding labels as inputs. Labels are supplied during training, so the latent vector can be associated with a specific label, establishing predictable image generation [41]. We used six classes selected as the positive data from the ISIC2019 for training and generating fake skin lesions. Samples from the positive and negative datasets are shown in Figure 3.

**Figure 4:**
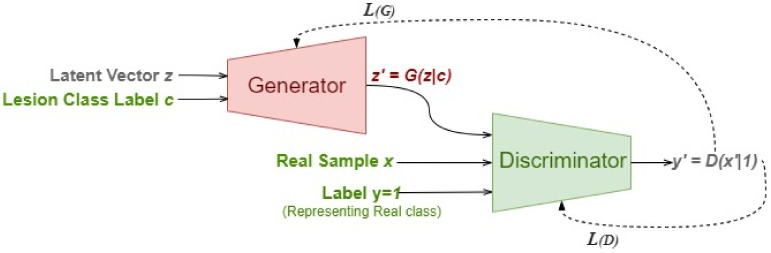
Derm-CGAN Framework.

Figure 4 illustrates the overall mechanism of the Derm-CGAN architecture and the design of the generator and the discriminator. Learning happens concurrently with the back-propagation of the generator and discriminator loss functions, even though each module of the framework remains independent of the others.

#### 2.2.1. The Conditioned Generator

**Figure 5:**
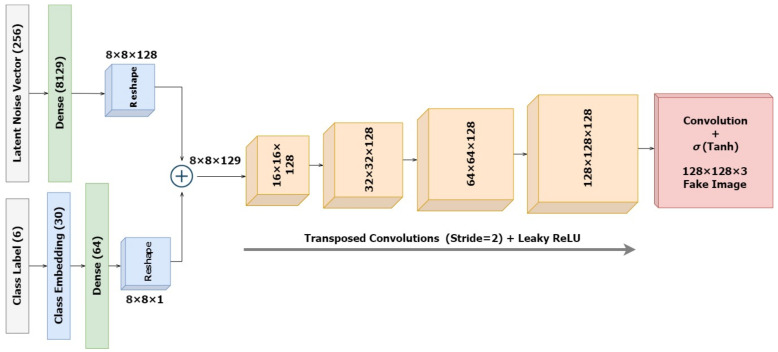
Architecture of the Conditioned Generator.

The architecture of the standalone generator model is designed as in Figure 5 that takes a latent noise vector of dimension 256 and a random label in the range [0, 5] representing the different skin lesion types. As a starting point, we reshape the latent input vector into an 8 × 8 image. For this, the latent vector is mapped to 128×8×8 = 8129 dense nodes, which are further reshaped into 8 × 8 images with 128 feature maps. The embedding of the category label inputs results in a vector of size 64 that will eventually be reshaped into an 8 × 8 image representation to fit the dimensions for concatenation with the reshaped latent noise vector. The combined vector size of 8 × 8 × 129 is slowly upscaled to a 128 × 128 × 3 image for output. Up to the output layer, this part of the cGAN is identical to an unconditional GAN. We integrate the input label and the latent input while defining the model inputs. Unlike the discriminator, this model is not explicitly trained, and thus, it is not compiled initially.

#### 2.2.2. The Discriminator

**Figure 6:**
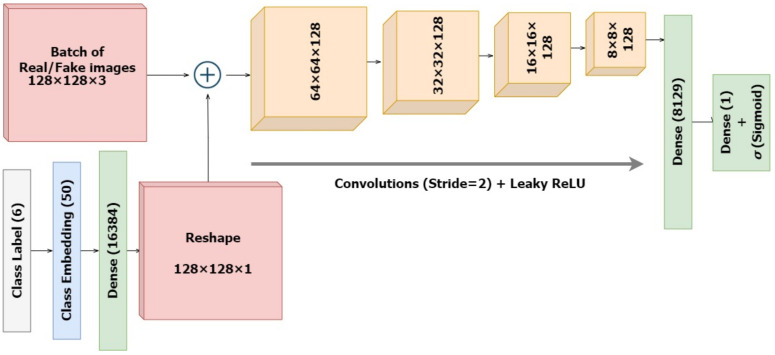
Architecture of the Discriminator Classifier.

The standalone discriminator model is designed as in Figure 6 to investigate the likelihood that the input image is real. Technically, it is a binary classifier deciding values between 1 and 0 using a sigmoid activation. Unlike regular GANs, here we are also providing several classes as input. The input images along with their skin lesion class labels are supplied as input to the discriminator. Similar to the generator, the discriminator also establishes embeddings for the class labels, which are then upscaled to the input image dimension of 128 × 128 with linear activations. Further, these are concatenated as an additional channel with the original input image. The classifier is designed to downsample the input embeddings of the combined representations four times, followed by a flattening layer and a dropout of 40% for regularization. Here again, we integrate the input label and image while defining the model inputs as performed in the generator. The sigmoid activation functions ensure sparse categorical values, representing real and fake data. The ground truth labels of the incoming images would always be set as real (y=1), regardless of whether they were batches of real or synthesized images. Eventually the generator tries to improve the possibility that the discriminator would misinterpret its inputs for genuine while the discriminator would want to accurately distinguish false as fake and real as real. However, the model is compiled before connecting it with the generator architecture.

#### 2.2.3. Derm-CGAN

The Dermatology-Conditioned Generative Adversarial Networks (Derm-CGAN) are assembled by taking noise and class labels, further synthesizing dermoscopic fakes, and outputting a classification for a batch of real and fake data. However, cGAN learns by juggling the training procedures of the generator and the discriminator separately. To update the generator, we combine the two networks and set the discriminator untrainable. At the same time, we keep the generator constant during the training of the discriminator. As a matter of fact, we will train the GAN on a half batch of real images and another half batch of fake images. We assign label 1 to real images and label 0 to false images.

Initially, we started the training of Derm-CGAN with the conditioned generator by pumping in noise vectors and skin class label inputs to produce 128 × 128 × 3 fake samples with the class label 0. We loop through a number of epochs to train our discriminator by first selecting a random batch of n real images from the real dataset. Further, a set of n images is produced from the still-learning generator. Both sets are fed to the discriminator to initiate training. Finally, the loss parameters are distinctly set and back-propagated for both the real and fake images (Equations (3) and (4)). Alongside this, a combined GAN loss comprehends the convergence rate of the framework.

(3) $$L_{\left(G\right)}=min\;\left[\begin{array}{c} \frac{1}{n}\sum_{i=1}^{n}\left(log\left(1-D\left(G\left(z_{i}|c\right)\right)\right)\right) \end{array}\right]$$

(4) $$L_{\left(D\right)}=max\;\left[\begin{array}{c} \frac{1}{n}\sum_{i=1}^{n}\left(logD\left(x_{i}|1\right)+log\left(1-D\left(G\left(z_{i}|c\right)\right)\right)\right) \end{array}\right]$$

Normally, the discriminator model is tuned for a single batch that consists of half real samples and half fake samples. However, we independently train the discriminator on either real or fake batches. The generator desires that the discriminator identify the samples it generates as legitimate samples. Since the generator is attempting to deceive the discriminator into believing the generated image is real at this point, we set the label as 1 (true/real). Hence, reversed labels are framed for the fake samples. The success of the discriminator lies in identifying the fake sample and classifying them to class 0 (fake). Instead, the output would be 1 (true) if the generator was successful in deceiving the discriminator. The generator error is hence updated using the discriminator loss.

#### 2.2.4. Representation Similarity Analysis

The conditioned generator assisted in creating images of uncommon skin disorders in minorities. This could diversify the datasets with respect to the subsequent skin condition fraud detection. We performed the Representation Similarity Analysis (RSA) on our generated data with real dermoscopic images. The computational approach of RSA is a technique of finding correlations between pairs of data to uncover their representation in a higher dimensional space [42].

We randomly selected 30 samples from each of the classes (real and fake) for the analysis. Each image was reshaped to a resolution of 200 × 200 and flattened. The rationale behind this is to project each image to a data point of dimension 40,000 for easier comparison. We find the Pearson correlation coefficients between all pairs of data points in the shared representational space. Equations (5) and (6) show the computations involved.

(5) $$\rho_{R^{D}}\left(x,y\right)=\frac{C_{xy}}{\sqrt{C_{xx}C_{yy}}}$$

(6) $$C_{xy}=\frac{1}{D}\sum_{i=1}^{D}\left(x_{i}-\bar{x}\right)\left(y_{i}-\bar{y}\right)$$

where the correlation coefficient is computed from the covariance between pairs of data points. These lie in the range of [−1, 1], with values closer to 1 (darker regions) implying a positive correlation. Here, it would mean higher similarity between pairs of data.

### 2.3. DeepFake Detection Architecture

We utilized the Vision Transformer (ViT) used in computer vision that operates inspired by the attention transformers used in Natural Language Processing (NLP) [43,44]. The transformer learns internally by assessing the relationship between input token pairs. It is a deep learning model that utilizes attention processes to weigh the relevance of each element of the input data separately.

**Figure 7:**
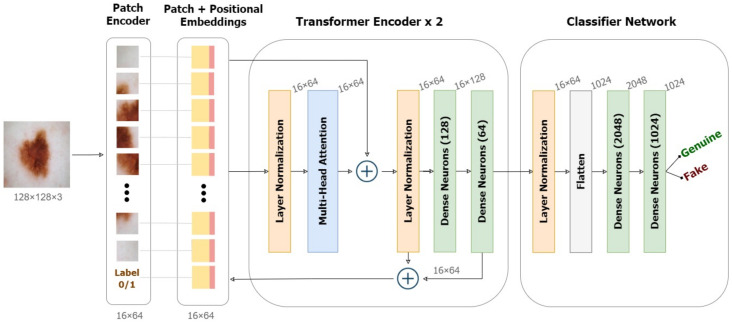
Framework of the Detection model based on Vision Transformers.

Figure 7 illustrates the working of ViT on deepfakes. Our model adheres as precisely as possible to the original Vision Transformers architecture. Patching, positional embeddings, and transformer encoders are the key components of a ViT.

In NLP transformers, a 1-dimensional series of token embeddings are processed to achieve tasks. Images are essentially 1 dimensional when converted to a series of flattened patches. Image patching is performed by separating the image into fixed-size parts, flattening them, and then linearly projecting them into a 2D data space (Equation (7)).

(7) $$X\epsilon R^{H\times W}\Rightarrow \;X_{p}\epsilon R^{N\times p^{2}}$$

Transformers utilize a clever positional encoding approach in which each position or index is mapped to a vector. As a result, the positional encoding layer produces a matrix in which each row represents one encoded object in the sequence aggregated with its positional information. To maintain positional information, position embeddings are added to patch embeddings. To the series of embedded patches, we append a learnable embedding for the class label (real/fake), whose state at the Transformer encoder’s output would serve as the representation y’ to be extrapolated using a classifier head.

Further, the transformer encoder receives the generated series of embedding vectors as input. Transformer encoders employ the self-attention layer allowing information to be embedded globally over the total picture. The model also learns from training data to encode the relative placement of image patches in order to recreate the image’s structure. Multiple instances of the self-attention layers, known as multi-head self-attention layers, linearly concatenate all attention outputs to the appropriate dimensions. This helps in the training of local and global dependencies in an image. The internal structure of the transformer encoder is illustrated in Figure 7. We used a pair of dense Multi-Layer Perceptrons (MLP) as the final classifier with the softmax activation.

Deepfake detection in itself is an exceedingly challenging task. Hence, we designed a ViT with a larger patch size from the original 128 × 128 resolution image data. Dosovitskiy et al. establish through various experiments on how the number of training parameters hugely reduces with smaller patch sizes while also maintaining the model performance on the task of deepfake detection [44]. However, we experimented with altering the input size and patch sizes without changing the overall structure of the architecture, analyzing the number of parameters all the time.

### 2.4. Evaluation

We report the Representation Similarity Matrix (RSM) using Pearson’s coefficient as a quantitative measure of the generation of the dermatological deepfake. Primarily, deepfake detection is a binary classification problem. We assessed the confusion matrix depicting the different blocks to which a predicted label could be applied. True positives and negatives are the numbers of real and fake classes that are rightly predicted, while false positives and negatives are those that are incorrectly predicted. With the components of the confusion matrix, the detection performance of the transformer classifier network has been analyzed in terms of accuracy, precision, recall, the Receiving Operating Characteristics (ROC), and Area under ROC curve (AUC) metrics. We chose the ROC curve over the PR curve due to the balanced selection of the dataset, albeit both are presented. The network was also computationally assessed in terms of the trainable parameters.

## 3. Results and Discussion

We discuss the two paradigms of deepfakes in a medical setting: generation and detection. Realistic dermoscopic skin lesions were synthesized using the proposed Derm-CGAN, and the detection of the same was approximated using the state-of-the-art Vision Transformers (ViT). We have consolidated the study with a comprehensive examination of the detection mechanism by evaluating the highest-performing variants of ViTs on the Diverse Fake Face Dataset (DFFD) and existing popular pre-trained deep architectures.

### 3.1. Dermoscopic Fake Generation

We initiated the training of Derm-CGAN by first generating points in the latent space for the generator. Further, the generator and discriminator were trained consecutively one after the other. For the discriminator, n fake samples were synthesized using the generator by feeding the latent vector of size 256 and labels for n samples. These, along with a random set of real images, were fed to the discriminator. The generator is then trained based on the discriminator loss. Likewise, the entire framework was run for 1200 epochs at a batch size of 32.

The total number of model parameters in our proposed model explains the complexity of this architecture. Table 2 projects the number of trainable and non-trainable parameters of the generator, discriminator, and the combined cGAN. The runtime and computational complexity of the designs are determined by the number of trainable parameters. Despite being built separately, the generator and discriminator designs go through intermittent training one after the other. It is noticeable that the total parameters of the GAN module correspond to the generator parameters and a pair of discriminator parameters (one each for real and fake sets). One set of discriminator weights is established as trainable throughout the passing of real data. At the same time, the weights corresponding to the fake data pass are made to be untrainable. The settings are reversed when the fake data pass is in effect.

**Figure 8:**
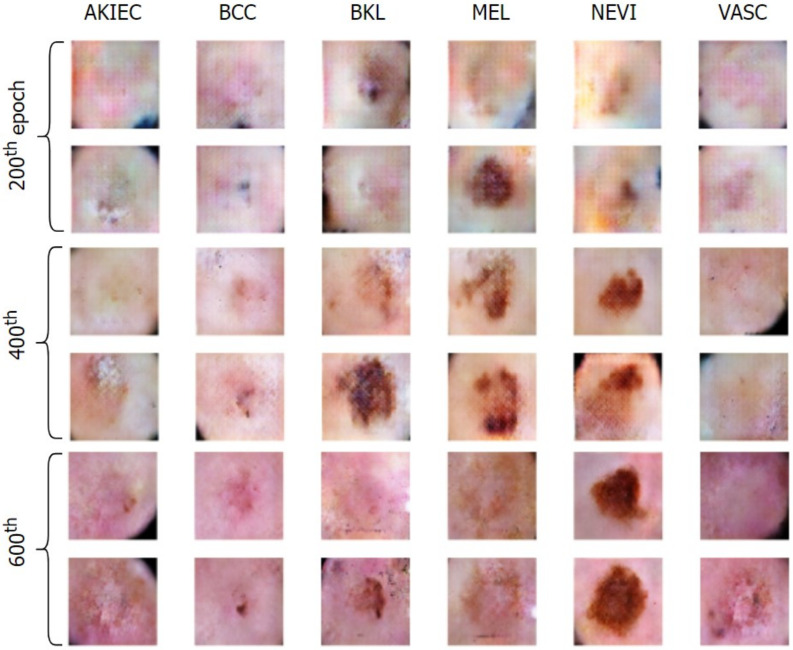
Generation of conditional skin lesions associated in each category at the initial 600 epochs.

**Figure 9:**
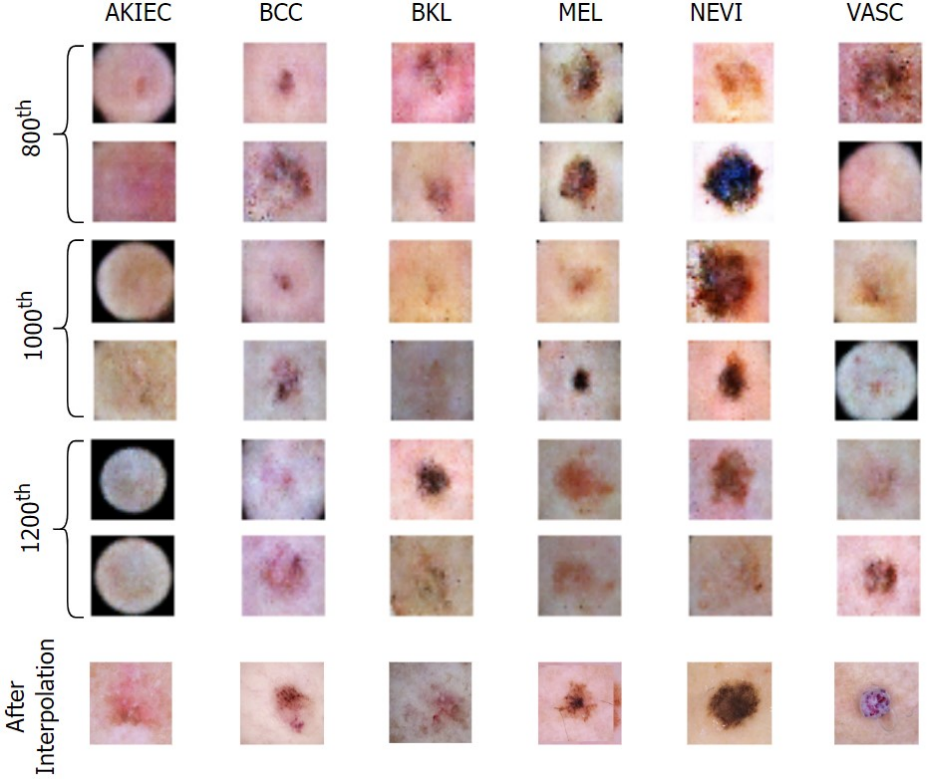
Generation of conditional skin lesions in each category at the final epochs.

Figure 8 and Figure 9 exhibit the progressive learning activity of the generator captured every 200 epochs. The conditional generator could seamlessly produce data points (fake dermatological conditions) based on the label corresponding to the skin lesion disease. It must be due to the human hair structures in the original image that the final synthesized images had a few missing pixel points (black dots). To remove the pixelated black noise, we performed localized interpolation based on thresholds on the outputs from the generator. The thresholds were set to near black point pixel values [0,0.1] in the normalized ranges.

**Figure 10:**
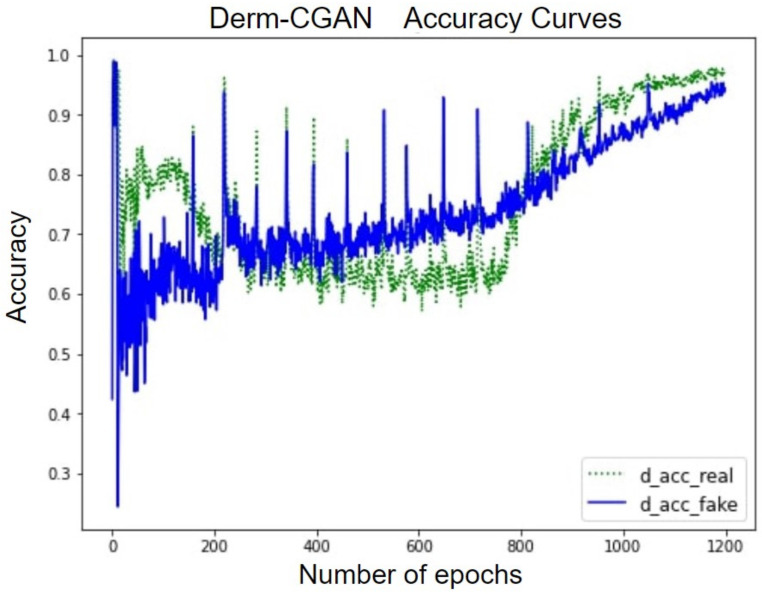
Accuracy curves of the discriminator on real and fake batches.

The accuracy curve of the discriminator over the real and fake classes helps explain the performance of Derm-CGAN (Figure 10). Though the learning process is quite disturbing, both detection accuracies have not gone below chance values after the initial 100 epochs. Furthermore, discriminating between real and fake has reached a tipping point in the final stages of learning. The accentuating curves demonstrate how the discriminator was able to clearly distinguish between the two classes. Both accuracies at the last epoch were estimated to be 98.43% and 96.79%.

**Figure 11:**
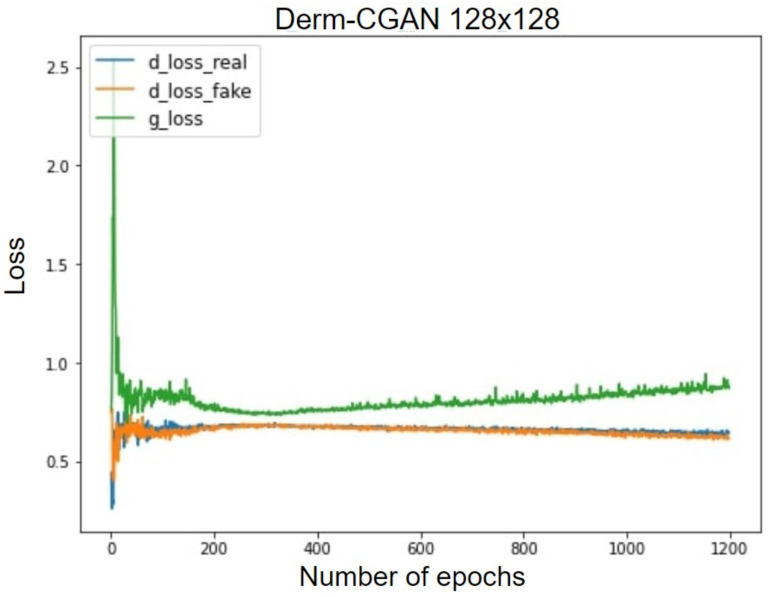
Generator and discriminator losses of Derm-CGAN.

Technically, the generator and discriminator should be competing against each other, and hence, when one improves, the other suffers bigger losses. The losses are negatively correlated. This happens until one or the other learns to minimize received losses more effectively. Figure 11 projects the GAN losses of producing realistic dermoscopic images. g_loss stands for the generator loss, whereas d_loss_real and d_loss_fake represent learning losses on real and fake batches separately.

The synthesized signal in the early epochs is very different from the real one, which results in good loss values for the d_loss_fake. The initial stages of realizing real images also pose the same difficulty. Due to the difficulty of the generator’s mission, it is initially challenging for it to identify a suitable gradient to follow during training. As a result, during the early training epochs, the generator loss exhibits rather unpredictable behavior. At about the 200th epoch, the generator begins to improve. This leads to the deterioration of the discriminator task performance as it becomes more difficult to classify. We also observed that after the 300th epoch, the discriminator losses are gradually and continuously decreasing, which is a further indication that the training strategy is effective. At the 1200th epoch, g_loss decreased to 0.82, d_loss_real to 0.54, and d_loss_fake to 0.52. GANs tend to fall to some minimal optima and reach a mode collapse. However, our framework eventually reached an optimal tradeoff of generator and discriminator losses.

**Figure 12:**
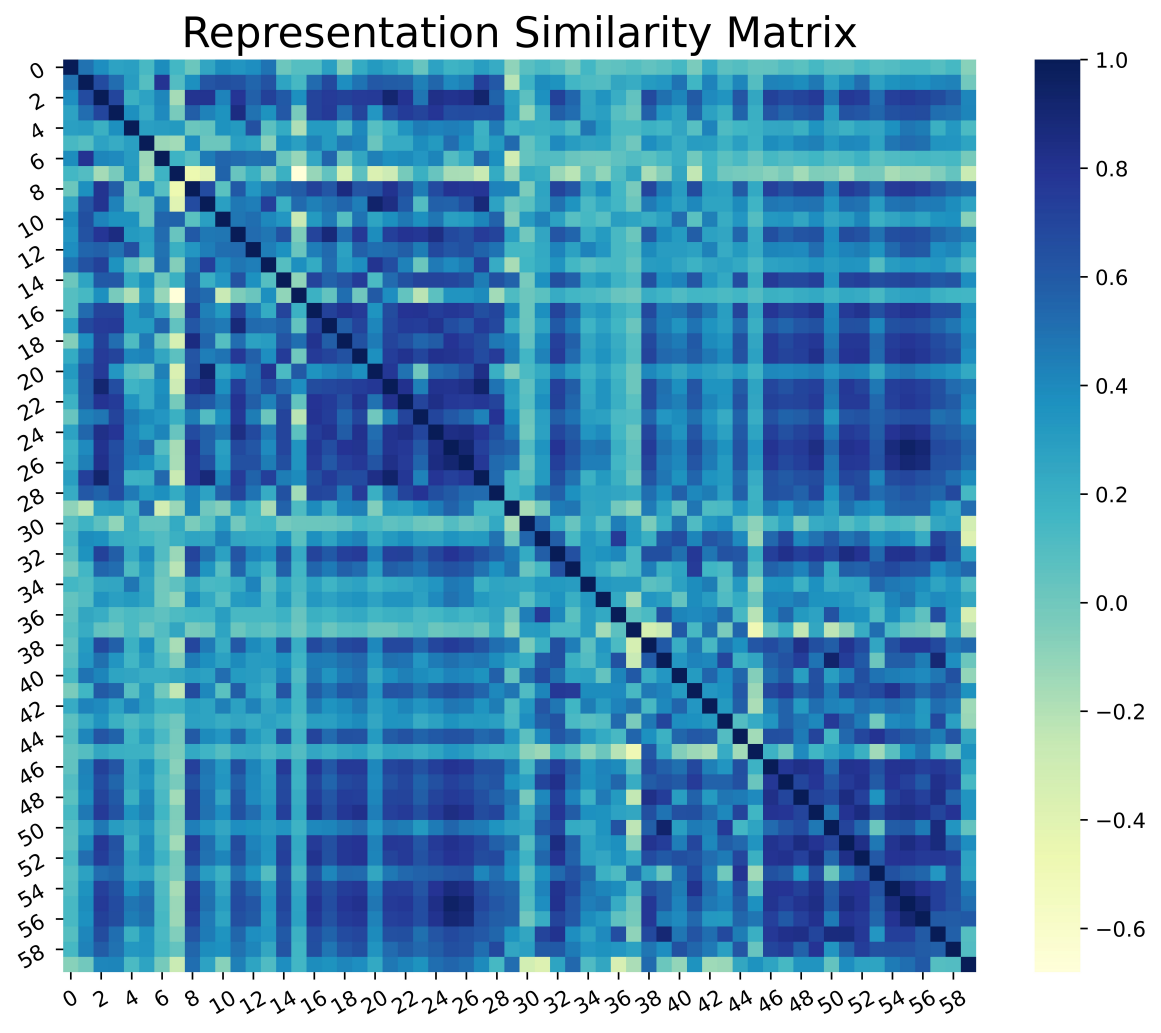
Representation Similarity Matrix of Real and Generated Skin lesions.

Representation Similarity Analysis was performed on real and synthesized skin lesion images by observing the Representation Similarity Matrix (RSM). Figure 12 illustrates the RSM of 30 random samples of each of the real and fake datasets. The initial 30 samples represent genuine data, whereas the last 30 belong to the fake class. The darker regions of RSM show how similar the images are in a higher dimensional space. The diagonal dark line represents a perfect correlation of data with itself. However, pairwise correlation presents a highly positive correlation (values close to 1) among most of the data points in the two class regions.

### 3.2. Dermoscopic Fake Detection

As observed from the RSM in Figure 12, deepfake detection would be an exceedingly challenging task. We conducted multiple experiments by varying the image reshape sizes and patch sizes that are to be input to the transformer encoder. The trainable number of parameters and other detection evaluation metrics were also assessed during the process. We have named the variants of ViT in the form of image size by patch size; for instance, ViT128/32 would mean the ViT settings with an input image size of 128 × 128 and patching size of 32 × 32. Table 3 projects the total number of trainable parameters for different settings of image and patch sizes. This would explain the complexity of utilizing a network model without forfeiting the performance of the same.

The patches per image depend solely on the input image size and the patch sizes. The elements per patch contribute to the weight parameters in the feature maps, which is the reason for the reduced number of parameters with respect to the elements per patch. It is also noticeable that the number of parameters decreases as we set conditions that eventually add to the model’s complexity.

**Figure 13:**
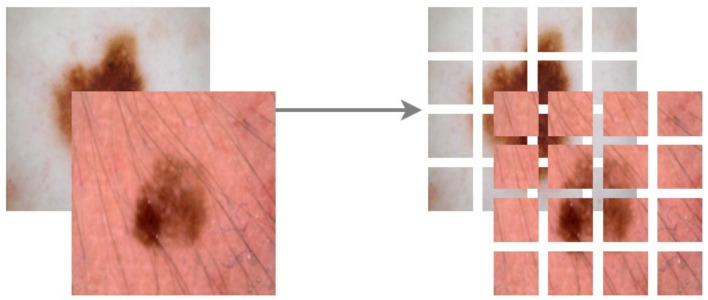
Patching Image size of 128 × 128 to 32 × 32 patches.

We omitted the ViT64/32 and the ViT32/16 variants, as patching would produce only 4 large patches. The rest of the model settings were assessed in terms of the aforementioned metrics. We trained the ViT frameworks for 100 epochs with a batch size of 10. The entire dataset was split for training and testing in the ratio 75:25. Of the 75% training data, 30% was randomly selected for validation in each epoch, thereby ensuring cross-validations during the training phase. Further, we applied Normalization, Random Horizontal Flip, Random Rotation of 20%, and Random Zoom of 20% augmentations to regularize our training on varied data. Each image was then patched depending on the ViT variant chosen. Figure 13 shows the patching of a random skin lesion on ViT128/32

The models were learned by minimizing the categorical cross-entropy cost function using the Adam optimizer in the default setting. Table 4 presents the performances of the ViT variants by fixating the hyper-parameters constant throughout.

Since the datasets are entirely balanced, the accuracy, precision, and recall fall in a similar range of values in each experiment. The ViT128/32 variant outperformed all other model tweaks at a margin of about 7%. However, ViT64/16 is similar to (but the best of) most other variants in the experiments. Hence, we assess the loss curves, ROC, and PR curves of these two experiments.

**Figure 14:**
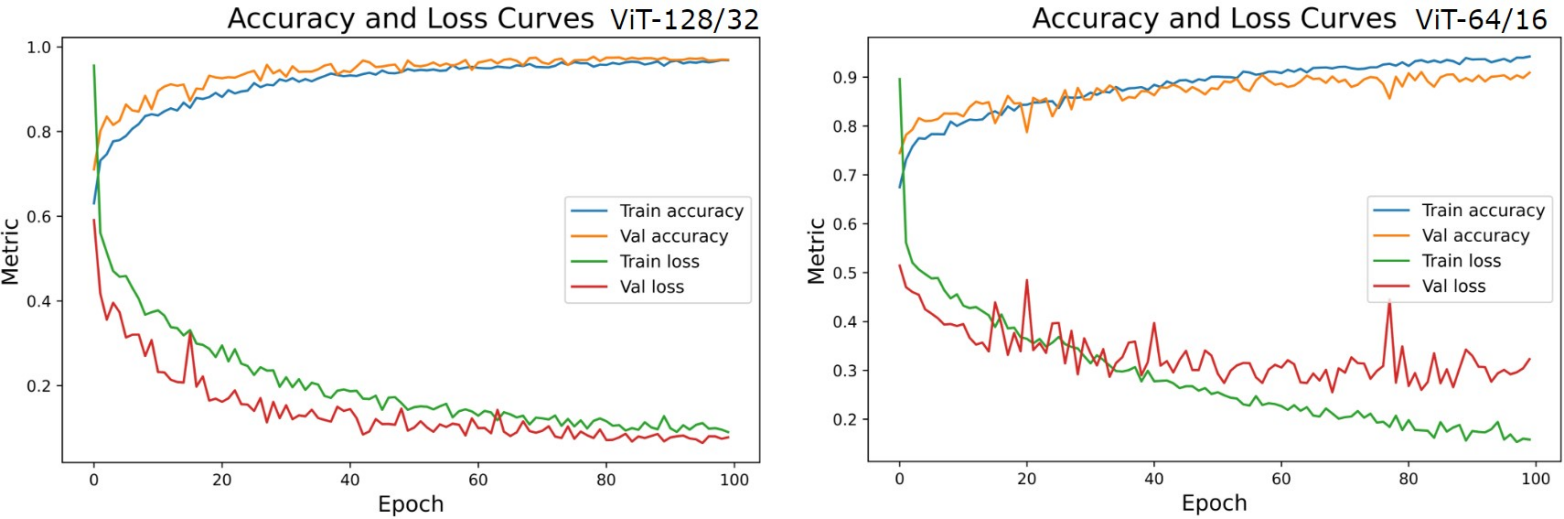
Accuracy and Loss curves of the two top performing model settings-ViT128/32 and ViT64/16.

Accuracies and losses during the network training were traced to deduce the behavior of the models during experiments (Figure 14). The training curves display the model’s ability to fit the training data. The tracked behavior of the validation data gives us an insight into how considerable the learning is on previously unknown data [45]. We observed that the training curves of all experiments were learned with near perfection. ViT128/32 has learned 97.34% of seen data and 96.78% of validation data, whereas ViT64/16 could capture about 95.21% of train data and could only approximate 86.38% of unseen fake and real images. In the loss curves of Figure 14, it was observed that the validation loss curves of ViT128/32 are consistent with the training curve, ruling out any chances of over-fitting. On the contrary, the loss curves of ViT64/16 imply clear overfitting of train data inferred at about the 40th epoch, after which the network could not improve further. This could be the reason for lesser test scores in the rest of the experiments.

**Figure 15:**
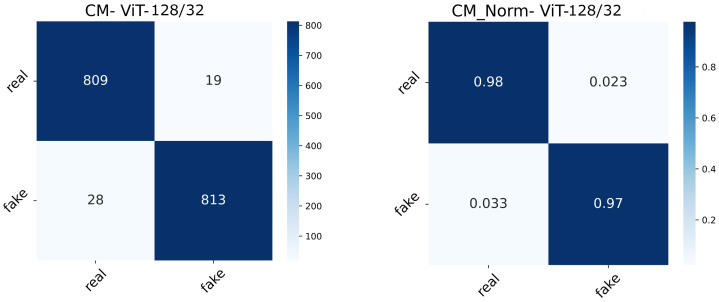
Confusion matrix (normal and normalized) of the ViT128/32 setting.

**Figure 16:**
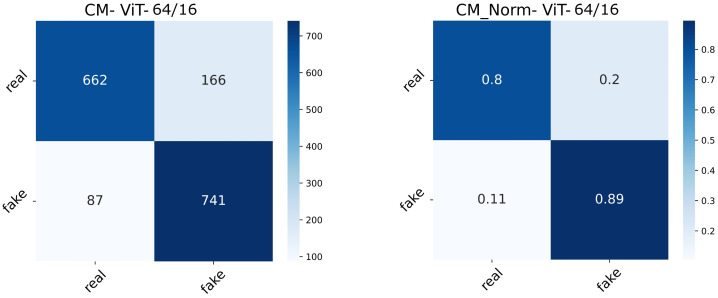
Confusion matrix (normal and normalized) of the ViT64/16 setting.

The real-valued and normalized confusion matrices of the two ViT variants show the proportion of each category classified (Figure 15 and Figure 16). The best-performing model could capture better attention representations, hence the higher percentages of True Positives and True Negatives. ViT64/16 has captured attentive features from the real image class, depicting a higher number of False Negatives (real being classified as fake) compared to False Positives (fake being classified as real).

**Figure 17:**
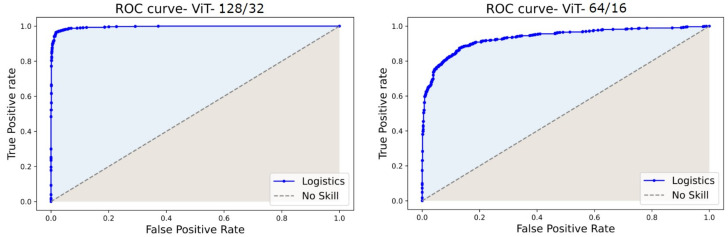
ROC curves of ViT128/32 and ViT64/16.

**Figure 18:**
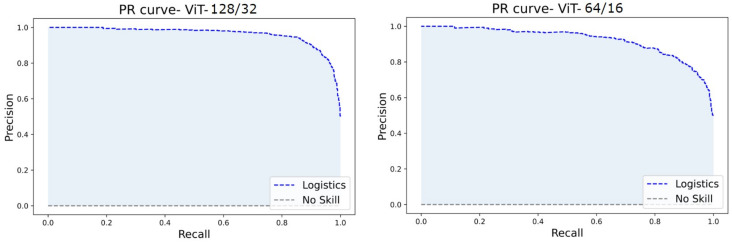
PR curves of ViT128/32 and ViT64/16.

The ROC and PR curves for the predictions at various categorization thresholds are illustrated in Figure 17 and Figure 18. The trade-off between the true positive rate and false positive rate was summarised by ROC curves, whereas the accommodation of the true positive rate and the predicted positives was summarized by the PR curve. The larger number of True Negatives by ViT64/16 causes the ROC and PR curves to fall at the *x*-axis. Moreover, the AUC sheds light on the competence of a predictive model. An AUC of 99.54% was computed for ViT128/32, and for other variants, it covered over 96.00% through 98.00%.

### 3.3. Discussion

The proposed model could conclude Vision Transformers to be effective on GAN-generated dermoscopic skin lesion deepfakes. There have been no other works conducted in this arena for comparison. However, we have compared the study with medical deepfake detection of data produced using Conditional GANs in Mirsky et al. [22], and the original Vision Transformer adaptations [44]. Further, we performed an ablation study by evaluating the best configuration of ViT128/32 on the Diverse Fake Face Dataset (DFFD). Table 5 summarizes the comparative study.

The detection accuracies of pre-trained networks in [34] and convolution neural networks in [35] on CT-GAN generated fake nodules could capture the underlying spatial artifacts inserted during the generation process through convolution operations. Furthermore, we observed that vision transformers have the potential to capture the best features from smaller datasets by enforcing attention wherever required. Dosovitskiy et al. [44], in their ablation study, inferred ViTs to perform well on smaller datasets. However, the number of parameters in their different variants exceeds over 86 M. The ViT/L represents a transformer with 24 layers and 16 multi-attention heads. The state-of-the-art ViT has performed well on the smaller dataset CIFAR10 comprising 6000 images, compared to CIFAR100, comprising 60,000 images. However, our proposed model stands close to the CIFAR10 experiment, with much lesser parameters (4.6 M) and only four multi-attention heads, leading to faster execution.

#### Critical Analysis

All models were trained and tested in Python 3.8.10 on NVIDIA Tesla V100-PCIE Graphics Processing Units (GPU) configured on a high-performance computing cluster with 1 Teraflop. The experiment is critically analyzed in two aspects: performance on other benchmark datasets and complexity compared to pre-trained convolution neural networks.

The model has been critically analyzed by utilizing the best-performing model ViT128/32 for fake face detection on the Diverse Fake Face Dataset (DFFD). DFFD comprises artificially synthesized and manipulated images using openly available generative methods. The data generated by PGGANs and StyleGANs were chosen as the negative data, and the Celeb-A dataset was chosen as the positive data for the ablation study. PGGAN dataset comprises 9975 train images and 8970 test images, whereas the stylegan dataset comprises 10,000 images in total, which were split in the ratio 60:40. However, Celeb-A contains 202,600 high-resolution images of which 10,000 were selected to maintain balance in the dataset. Table 6 projects the performance of ViT128/32 on the selected datasets. The performance degradation in StyleGAN-generated images would be due to the highly realistic nature of the synthesized data. Nevertheless, the optimized settings of the proposed ViT128/32 could perform comparably to each other. The training time for the two fake face experiments took ∼17 s/epoch, whereas the proposed model trained in ∼13 s/epoch. Test runtimes were very quick as they were processed in batches of 8 and took ∼5–8 ms.

The space complexity of the proposed ViT model was estimated by computing the number of trainable parameters with respect to popular pre-trained networks. As estimated from Table 3, the ViT128/32 has required 4.6 M parameters compared to the other variants. We trained and tested the best pre-trained models from the literature on this task, all the time keeping track of the training time and the trainable number of parameters. Table 7 shows the estimated complexities and performances of the pre-trained models. The pre-trained models exhibit faster runtimes compared to ViTs as they are composed using convolution layers. However, ViTs, with their few numbers of layers, lesser trainable parameters, and no convolution layers, exhibit comparatively better performance. Interestingly, the depth of the pre-trained convolutional neural networks was directly proportional to the categorization performance. Furthermore, it is observed that the ViT could capture the hidden dynamics of the GAN traces injected during the synthesis process.

Technically, it is easier to insert fakes into the dermoscopic imaging modality as they are non-invasively captured. This would be the reason for the forthcoming potential of generating dermoscopic deepfakes. Our work is limited to the dermoscopic avenue of healthcare. There is a huge scope for deepfake generation in the various 2D and 3D imaging modalities such as X-rays, MRIs, or f-MRIs. This arena of medical deepfakes is relatively new, with very few published studies to compare with. Anybody could use faking technology to benefit from insurance fraud or cause harm through medical misdiagnosis. Nevertheless, such a detection technology would assist non-specialists in detecting fraudulent attacks against them. Deploying the technique via a smartphone application would be advantageous for the general public.

## 4. Conclusions

Medical deepfakes are an open research domain that has acquired far too little emphasis and requires more attention in the research community. We experimented with synthesizing fake dermoscopic skin cancerous lesions using a label-conditioned GAN framework named Derm-CGAN. The realism of the generated dermoscopic deepfakes was analyzed using a Similarity Matrix. The same was then detected utilizing several variants of the state-of-the-art ViTs obtaining an optimized parameter setting for future research purposes. The proposed model has also been critically studied in terms of complexities and runtimes by comparing it with pre-trained detection models, benchmark datasets as well as the original results of ViTs. The development of detecting algorithms is still in its early phases, and a large pool of technical aspects could be explored. In the future, more signal-processing techniques could be employed in this domain. Future advancements in detection may also use more complex designs that could capture the inherent and hidden but explainable dynamics of data. Locating the region of interest where the fake has occurred is another avenue for more inquiry. Keeping aside the technicality of generation and detection of highly realistic fakes in the healthcare sector, we emphasize the societal impacts of such technology outbursts into the wild.

## Figures and Tables

**Table 2:** Number of trainable and non-trainable parameters in Derm-CGAN.

	Total Parameters (Millions)	Trainable	Non-Trainable
**Generator**	1.90 M	1.90 M	0
**Discriminator**	1.29 M	1.29 M	0
**GAN Module**	4.48 M	3.18 M	1.29 M

**Table 3:** Conditional settings of Vision Transformer.

Model Variant	Image Size	Patch Size	Parameters (Millions)	Patches	Elements/Patch
ViT128/32	128 × 128	32 × 32	4.6 M	16	3072
ViT128/16	128 × 128	16 × 16	10.7 M	64	768
ViT128/8	128 × 128	8 × 8	35.1 M	256	192
ViT64/32	64 × 64	32 × 32	2.9 M	4	3072
ViT64/16	64 × 64	16 × 16	4.4 M	16	768
ViT64/8	64 × 64	8 × 8	10.7 M	64	192
ViT32/16	32 × 32	16 × 16	2.8 M	4	768
ViT32/8	32 × 32	8 × 8	4.4 M	16	192
ViT32/4	32 × 32	4 × 4	10.7 M	64	48

**Table 4:** Performance of the ViT variants.

Model	Accuracy (%)	Precision (%)	Recall (%)	F1 Score (%)	AUC (%)
**ViT128/32**	**97.18**	**96.65**	**97.70**	**97.19**	**99.54**
ViT 128/16	88.92	89.65	88.91	88.87	96.13
ViT 128/8	86.22	87.37	86.21	86.12	93.40
**ViT 64/16**	**89.85**	**89.87**	**89.86**	**89.85**	**96.56**
ViT 64/8	88.86	89.96	88.86	88.79	96.27
ViT 32/16	85.02	85.03	85.02	85.02	92.18
ViT 32/8	83.21	83.59	83.21	83.16	92.14

**Table 5:** Comparative study on similar Datasets and Detection Model Performances.

Method	Dataset	Accuracy (%)	Sensitivity (%)	Specificity (%)
DenseNet [34]	CT-GAN	80.40	71.30	71.75
3DCNN [35]	CT-GAN	91.57	91.42	97.20
ViT-L/16 [44]	CIFAR100	93.90 ± 0.05	Not Specified	Not Specified
ViT-L/16 [44]	CIFAR10	99.42 ± 0.03	Not Specified	Not Specified
Proposed	Derm-CGAN	97.18	97.70	96.76

**Table 6:** Performance Analysis of ViT128/32 on DFFD benchmark data.

Dataset	Accuracy (%)	Precision (%)	Recall (%)	F1 Score (%)	AUC (%)
DFFD (PGGAN) vs. Celeb-A	96.76	96.81	96.33	96.73	99.48
DFFD (StyleGAN) vs. Celeb-A	89.81	91.95	85.16	89.79	96.13
Derm-CGAN vs. ISIC2019	97.18	96.65	97.70	97.19	99.54

**Table 7:** Complexity Comparison of Derm-CGAN generated data on pre-trained and proposed architectures.

Model	Parameters (Millions)	Number of Layers	Runtime (s)	Accuracy (%)	Precision (%)	Recall (%)
VGG16	17.9 M	20	400	55.30	30.59	55.30
ResNet50	24.8 M	54	900	71.20	72.73	71.22
DenseNet	11.2 M	125	1000	85.09	86.63	83.74
EfficientNetB0	8.7 M	241	600	84.74	87.18	83.12
ViT128/32	4.6 M	30	1300	97.18	96.65	97.70

## Data Availability

The positive dataset is available at https://challenge.isic-archive.com/ (accessed on 19 November 2022). The negative dataset will be made available on request at https://www.mirworks.in/ (accessed on 19 November 2022).

## References

[1] Suwajanakorn S., Seitz S.M., Kemelmacher-Shlizerman I. (2017). Synthesizing obama: Learning lip sync from audio. ACM Trans. Graph. (ToG).

[2] Kietzmann J., Lee L.W., McCarthy I.P., Kietzmann T.C. (2020). Deepfakes: Trick or treat?. Bus. Horizons.

[3] Aswathy A., Anand H.S., Chandra S. (2022). COVID-19 severity detection using machine learning techniques from CT-images. Evol. Intell..

[4] Aswathy A., Vinod Chandra S. (2022). Detection of Brain Tumor Abnormality from MRI FLAIR Images using Machine Learning Techniques. J. Inst. Eng. (India) Ser. B.

[5] Misaj S., Vinod Chandra S.S. (2023). Detecting skin lesions fusing handcrafted features in image network ensembles. Multimed. Tools Appl..

[6] Srinivasu P.N., SivaSai J.G., Ijaz M.F., Bhoi A.K., Kim W., Kang J.J. (2021). Classification of skin disease using deep learning neural networks with MobileNet V2 and LSTM. Sensors.

[7] Ali S., El-Sappagh S., Ali F., Imran M., Abuhmed T. (2022). Multitask Deep Learning for Cost-Effective Prediction of Patient’s Length of Stay and Readmission State Using Multimodal Physical Activity Sensory Data. IEEE J. Biomed. Health Inform..

[8] Seow J.W., Lim M.K., Phan R.C.W., Liu J.K. (2022). A comprehensive overview of Deepfake: Generation, detection, datasets, and opportunities. Neurocomputing.

[9] Gaur L., Arora G.K., Jhanjhi N.Z. (2022). Deep Learning Techniques for Creation of DeepFakes. DeepFakes.

[10] Goodfellow I., Pouget-Abadie J., Mirza M., Xu B., Warde-Farley D., Ozair S., Courville A., Bengio Y. (2020). Generative adversarial networks. Commun. ACM.

[11] Chandra S., Hareendran S. (2021). Machine Learning: A Practitioner’s Approach.

[12] Korshunova I., Shi W., Dambre J., Theis L. Fast face-swap using convolutional neural networks. Proceedings of the IEEE International Conference on Computer Vision.

[13] Natsume R., Yatagawa T., Morishima S. (2018). Rsgan: Face swapping and editing using face and hair representation in latent spaces. arXiv.

[14] Li L., Bao J., Yang H., Chen D., Wen F. (2019). Faceshifter: Towards high fidelity and occlusion aware face swapping. arXiv.

[15] Kim H., Garrido P., Tewari A., Xu W., Thies J., Niessner M., Pérez P., Richardt C., Zollhöfer M., Theobalt C. (2018). Deep video portraits. ACM Trans. Graph. (TOG).

[16] Liu M.Y., Tuzel O. Coupled generative adversarial networks. Proceedings of the Advances in Neural Information Processing Systems 29 (NIPS 2016).

[17] Karras T., Aila T., Laine S., Lehtinen J. (2017). Progressive growing of gans for improved quality, stability, and variation. arXiv.

[18] Karras T., Laine S., Aila T. A style-based generator architecture for generative adversarial networks. Proceedings of the IEEE/CVF Conference on Computer Vision and Pattern Recognition.

[19] Brock A., Donahue J., Simonyan K. (2018). Large scale GAN training for high fidelity natural image synthesis. arXiv.

[20] Frid-Adar M., Diamant I., Klang E., Amitai M., Goldberger J., Greenspan H. (2018). GAN-based synthetic medical image augmentation for increased CNN performance in liver lesion classification. Neurocomputing.

[21] Thambawita V., Isaksen J.L., Hicks S.A., Ghouse J., Ahlberg G., Linneberg A., Grarup N., Ellervik C., Olesen M.S., Hansen T. (2021). DeepFake electrocardiograms using generative adversarial networks are the beginning of the end for privacy issues in medicine. Sci. Rep..

[22] Mirsky Y., Mahler T., Shelef I., Elovici Y. {CT-GAN}: Malicious Tampering of 3D Medical Imagery using Deep Learning. Proceedings of the 28th USENIX Security Symposium (USENIX Security 19).

[23] Douzas G., Bacao F. (2018). Effective data generation for imbalanced learning using conditional generative adversarial networks. Expert Syst. Appl..

[24] Rubin M., Stein O., Turko N.A., Nygate Y., Roitshtain D., Karako L., Barnea I., Giryes R., Shaked N.T. (2019). TOP-GAN: Stain-free cancer cell classification using deep learning with a small training set. Med Image Anal..

[25] Islam J., Zhang Y. (2020). GAN-based synthetic brain PET image generation. Brain Inform..

[26] Levine A.B., Peng J., Farnell D., Nursey M., Wang Y., Naso J.R., Ren H., Farahani H., Chen C., Chiu D. (2020). Synthesis of diagnostic quality cancer pathology images by generative adversarial networks. J. Pathol..

[27] Mangaokar N., Pu J., Bhattacharya P., Reddy C.K., Viswanath B. Jekyll: Attacking medical image diagnostics using deep generative models. Proceedings of the 2020 IEEE European Symposium on Security and Privacy (EuroS&P).

[28] Brian D., Joanna B., Ben P., Jikuo L., Russ H., Menglin W., Cristian C.F. (2020). The DeepFake Detection Challenge Dataset. arXiv.

[29] Dang H., Liu F., Stehouwer J., Liu X., Jain A. On the Detection of Digital Face Manipulation. Proceedings of the IEEE Computer Vision and Pattern Recognition.

[30] Rossler A., Cozzolino D., Verdoliva L., Riess C., Thies J., Nießner M. Faceforensics++: Learning to detect manipulated facial images. Proceedings of the IEEE/CVF International Conference on Computer Vision.

[31] Coccomini D.A., Messina N., Gennaro C., Falchi F. (2022). Combining efficientnet and vision transformers for video deepfake detection. Proceedings of the Image Analysis and Processing–ICIAP 2022: 21st International Conference.

[32] Lamichhane B., Thapa K., Yang S.H. (2022). Detection of Image Level Forgery with Various Constraints Using DFDC Full and Sample Datasets. Sensors.

[33] Suganthi S., Ayoobkhan M.U.A., Bacanin N., Venkatachalam K., Štěpán H., Pavel T. (2022). Deep learning model for deep fake face recognition and detection. PeerJ Comput. Sci..

[34] Solaiyappan S., Wen Y. (2022). Machine learning based medical image deepfake detection: A comparative study. Mach. Learn. Appl..

[35] Sharafudeen M., Vinod Chandra S. (2023). Medical Deepfake Detection using 3-Dimensional Neural Learning. Proceedings of the IAPR Workshop on Artificial Neural Networks in Pattern Recognition.

[36] Khan S., Naseer M., Hayat M., Zamir S.W., Khan F.S., Shah M. (2022). Transformers in vision: A survey. ACM Comput. Surv. (CSUR).

[37] Mirza M., Osindero S. (2014). Conditional generative adversarial nets. arXiv.

[38] Tschandl P., Rosendahl C., Kittler H. (2018). The HAM10000 dataset, a large collection of multi-source dermatoscopic images of common pigmented skin lesions. Sci. Data.

[39] Combalia M., Codella N.C., Rotemberg V., Helba B., Vilaplana V., Reiter O., Carrera C., Barreiro A., Halpern A.C., Puig S. (2019). Bcn20000: Dermoscopic lesions in the wild. arXiv.

[40] Codella N.C., Gutman D., Celebi M.E., Helba B., Marchetti M.A., Dusza S.W., Kalloo A., Liopyris K., Mishra N., Kittler H. Skin lesion analysis toward melanoma detection: A challenge at the 2017 international symposium on biomedical imaging (isbi), hosted by the international skin imaging collaboration (isic). Proceedings of the 2018 IEEE 15th International Symposium on Biomedical Imaging (ISBI 2018).

[41] Raji C., Anand H., Chandra S.V. (2017). Computer based prognosis model with dimensionality reduction and validation of attributes for prolonged survival prediction. Inform. Med. Unlocked.

[42] Anand H., Vinodchandra S. Applying correlation threshold on Apriori algorithm. Proceedings of the 2013 IEEE International Conference ON Emerging Trends in Computing, Communication and Nanotechnology (ICECCN).

[43] Vaswani A., Shazeer N., Parmar N., Uszkoreit J., Jones L., Gomez A.N., Kaiser Ł., Polosukhin I. (2017). Attention is all you need. arXiv.

[44] Dosovitskiy A., Beyer L., Kolesnikov A., Weissenborn D., Zhai X., Unterthiner T., Dehghani M., Minderer M., Heigold G., Gelly S. (2020). An image is worth 16 × 16 words: Transformers for image recognition at scale. arXiv.

[45] Anand H.S., Vinod Chandra S.S. (2016). Association rule mining using treap. Int. J. Mach. Learn. Cybern..

